# 1.27 kW, 2.2 GHz pseudo-random binary sequence phase modulated fiber amplifier with Brillouin gain-spectrum overlap

**DOI:** 10.1038/s41598-019-57408-5

**Published:** 2020-01-20

**Authors:** Meizhong Liu, Yifeng Yang, Hui Shen, Jingpu Zhang, Xingxing Zou, Hanbin Wang, Lucheng Yuan, Yang You, Gang Bai, Bing He, Jun Zhou

**Affiliations:** 1 Shanghai Key Laboratory of All Solid-State Laser and Applied Techniques, Shanghai Institute of Optics and Fine Mechanics, Chinese Academy of Sciences, Shanghai, 201800 China; 2 Center of materials Science and Optoelectronics Engineering, University of Chinese Academy of Sciences, Beijing, 100049 China; 3 Department of Physics, Harbin Institute of Technology, Harbin, 150001 China

**Keywords:** Lasers, LEDs and light sources, Fibre lasers

## Abstract

We present a 2.2 GHz modulated, 1.27 kW output power, monolithic fiber amplifier based on pseudo-random binary sequence (PRBS) phase modulation. The spectral line spacing of maximizing the threshold enhancement factor (plateau of trend) was found by theoretical simulation. The spectral line spacing was adjusted to 12.7 MHz by a pattern length of n = 9, which is close to the plateau of trend in the proposed architecture. A 2.2 GHz low-pass radio frequency filter was used to control the FWHM of the seed. A four-stage Yb-doped fiber amplifier chain was established to boost a distributed Bragg reflector (DBR) laser and a distributed feedback (DFB) diode laser to 1.2 kW and 1.27 kW with a backward reflectively of <1‰, which shows a good suppression of SBS effect.

## Introduction

Stimulated Brillouin scattering (SBS) is one of the leading nonlinear impairments in high-power narrow-linewidth continuous-wave (CW) fiber amplifiers^1^. In SBS process, optical power is transferred from the signal light and into the backscattered Stokes light^2^. Sufficiently high optical power initiates backscattered pulsation, which can severe damage the fiber amplifier. In order to suppress SBS and achieve higher laser power, phase modulation techniques have been implemented by fiber-coupled LiNbO_3_ electro-optic modulators. Phase modulation techniques include driving the phase modulator with a sine wave^3^, a flat-top spectrum radio frequency (RF) signal^4,5^, or a white noise source (WNS)^6^ etc. Among these linewidth broadening methods, >20 GHz linewidth can be generally obtained. Unfortunately, such linewidth may cause harmful effects for many fields, such as the beam quality degradation in spectral beam combining due to dispersion. To further suppress SBS at a much narrow level, a PRBS phase modulation technique has been theoretically and experimentally studied^7,8^. The advantage of PRBS has been proven in the long-distance optical communication systems^9^. PRBS generates an equally spaced discrete optical power spectral density, and the spectral line spacing is a function of the modulation frequency and pattern length, which can make the spectral line spacing adjustment flexible^10^. In the narrow linewidth fiber amplifier configuration, a proper spectral line spacing improves the SBS suppression for a given linewidth^11^. When the spectral line spacing is large enough, each discrete spectral component separately seeds the SBS process likes sine-wave modulation format^12^, and the SBS threshold is determined by the component with the highest spectral power. As the spectral line spacing is reduced, the greater overlap between the gain spectra of adjacent lines, and the concomitant enhancement of cross-interactions is demonstrated^11^. For a given linewidth, the number of spectral line is inversely proportional to the spectral line spacing. Enhancement of the SBS threshold cannot be achieved by simply increasing the number of spectral line. Therefore, exploring the proper spectral line spacing is key points to further scale laser power at a much narrow linewidth.

In this paper, we applied PRBS phase modulation with a 2.2 GHz low-pass RF filter to control the longitudinal mode spacing of the single frequency master oscillator, aimed to equilibrate spectral homogenization and Brillouin gain-spectrum overlap in the narrow linewidth fiber amplifier. The spectral line spacing of the phase modulated seed is chosen to be 12.7 MHz, which approach the plateau of the SBS threshold. A four-stage, 1.2 kW monolithic fiber amplifier is proposed by using a 7 m-long non-polarization maintaining 20/400 μm Yb-doped fiber, which is used to boost a DFB diode laser and a DBR fiber laser to 1.27 kW and 1.2 kW, respectively. The backward reflectively is measured to be <1‰. To the best of our knowledge, this is the highest power level for narrow linewidth fiber amplifier based on phase control under 5 GHz spectral linewidth.

## Results

**Figure 1:**
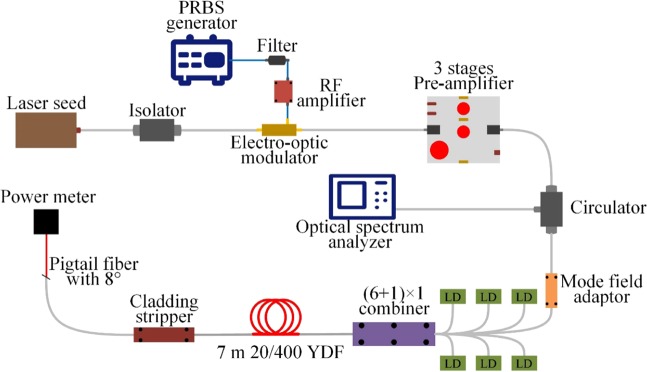
Schematic of the PRBS modulated, low pass filtered, 1.2 kW four-stage monolithic fiber amplifier.

**Figure 2:**
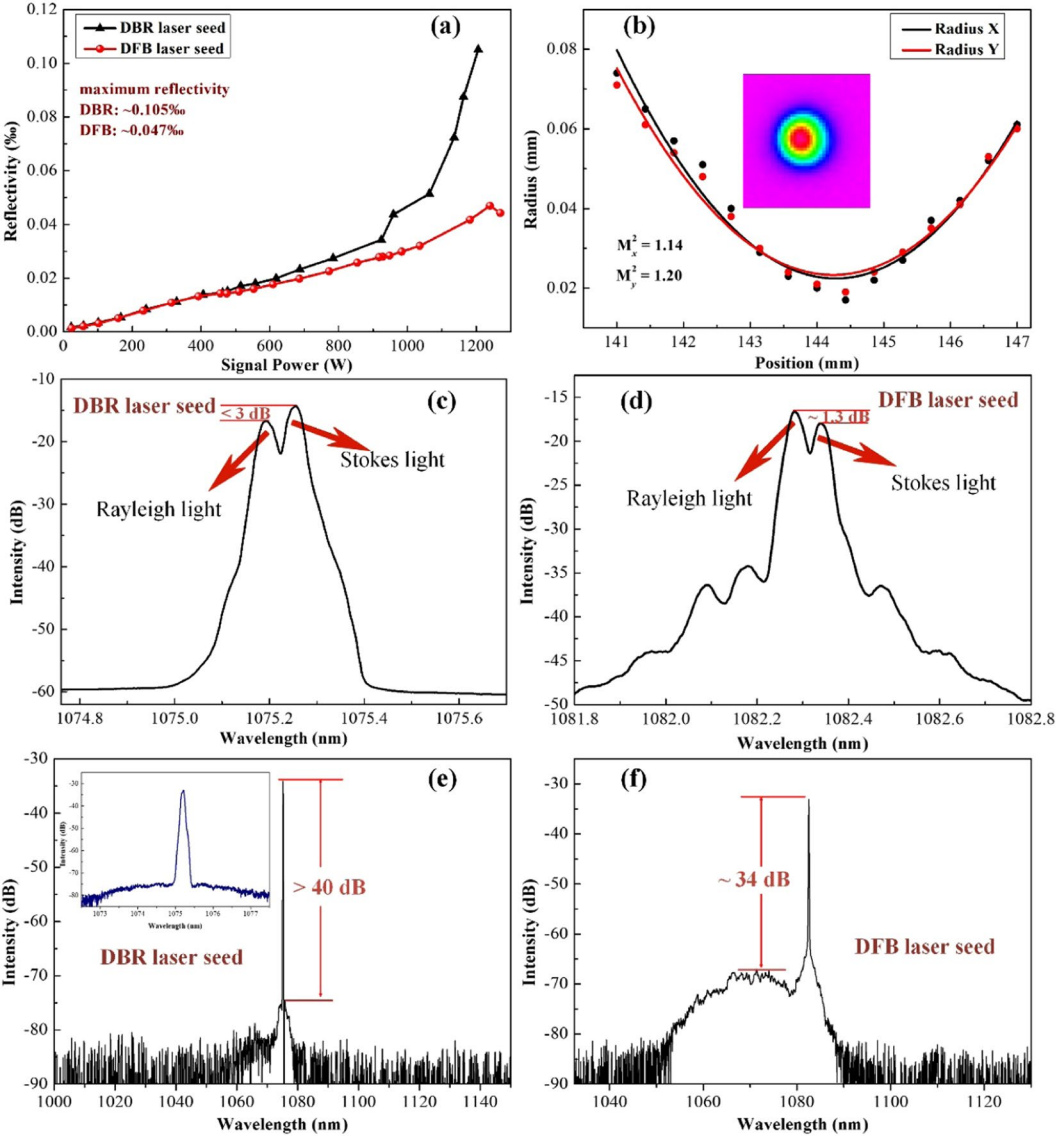
Fiber amplifiers output character of the DBR laser and the DFB diode laser seeding. (**a**) reflectivity as functions of DFB laser-seeded and DBR laser-seeded fiber amplifiers output power; (**b**) the measurements (dots) and fitting results (solid lines) of beam quality at the output power of 1 kW (**c**) backward light at maximum power for the DBR seeding with 6.5 GHz, PRBS9 phase modulation; (**d**) backward light at maximum power for the DFB seeding with 6.5 GHz, PRBS9 phase modulation; (**e**) forward spectrum of the amplifier operating at 1075 nm at maximum power (insert: narrow spectrum of 5 nm). (**f**) forward spectrum of the amplifier operating at 1082 nm at maximum power.

We established a PRBS modulated, low pass filtered, kilowatt-class, monolithic fiber amplifier. The proposed architecture is shown in Fig. 1. Reflectivity versus output power with DBR and DFB laser seed are shown in Fig. 2(a). As shown, signal power of 1.27 kW and 1.2 kW were attained at DFB and DBR laser seeding with near diffraction-limited beam quality (M^2^ < 1.2), respectively. In both cases, the reflectivity was much less than 1‰ of the previous amplifiers^1,13,14^, there is potential room for further power scaling. This work demonstrates the highest output power in <5 GHz regime based on phase-modulated approach. The DFB diode laser seed provided superior SBS suppression (lower reflectivity) in the same amplifier. Figure 2(c) shows that a characteristic of relative intensity of backward Rayleigh and Stokes light was utilized to identify the SBS threshold (with the former lower ~3 dB) in our experiment. The DFB laser-seeded amplifier obtained a weaker backward Stokes light than Rayleigh light at pump full power output as plotted in Fig. 2(d). The forward spectral content for fiber amplifier configurations were measured as shown in Fig. 2(e,f), the DBR laser-seeded amplifier provided an ASE suppression of more than 40 dB at full power output, while the DFB laser-seeded amplifier was characterized by an obviously ASE noise. The single frequency threshold of ~24 W was measured by using the same SBS threshold criterion, and the enhancement factor of ~53 was attained at maximum power in our experiment.

**Figure 3:**
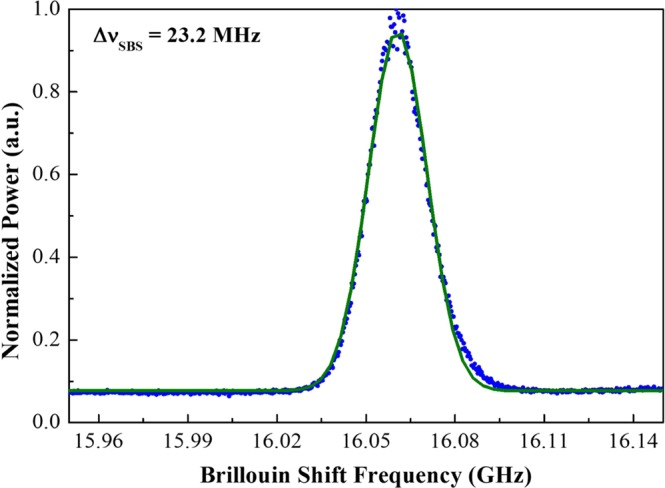
Brillouin gain spectrum of this fiber amplifier attained from self-heterodyne and estimated via Lorentzian lineshape fitting (solid curve).

A 1.2 kW four-stage fiber amplifier was used to explore proper spectral line spacing under PRBS modulation. The self-heterodyne technique^15^ was applied to characterize the Brillouin gain spectrum, and the measured spectral data of the amplifier were fitted with a Lorentzian lineshape to be a FWHM bandwidth of ~23.2 MHz, which is shown in Fig. 3. The estimated result of the Brillouin gain spectrum bandwidth is expectedly less than the Brillouin spontaneous bandwidth (~40–50 MHz) due to gain narrowing^1,7^. Figure 6(b) compares the measurement results of the amplifier SBS threshold when PRBS signal generator at eleven different spectral line spacings. The SBS threshold increases as the spectral line spacing decreases due to the spectral homogenization as presented in Fig. 7, and the saturation characteristic is reached when the spectral line spacing is reduced to about 10 MHz.

## Discussion

**Figure 4:**
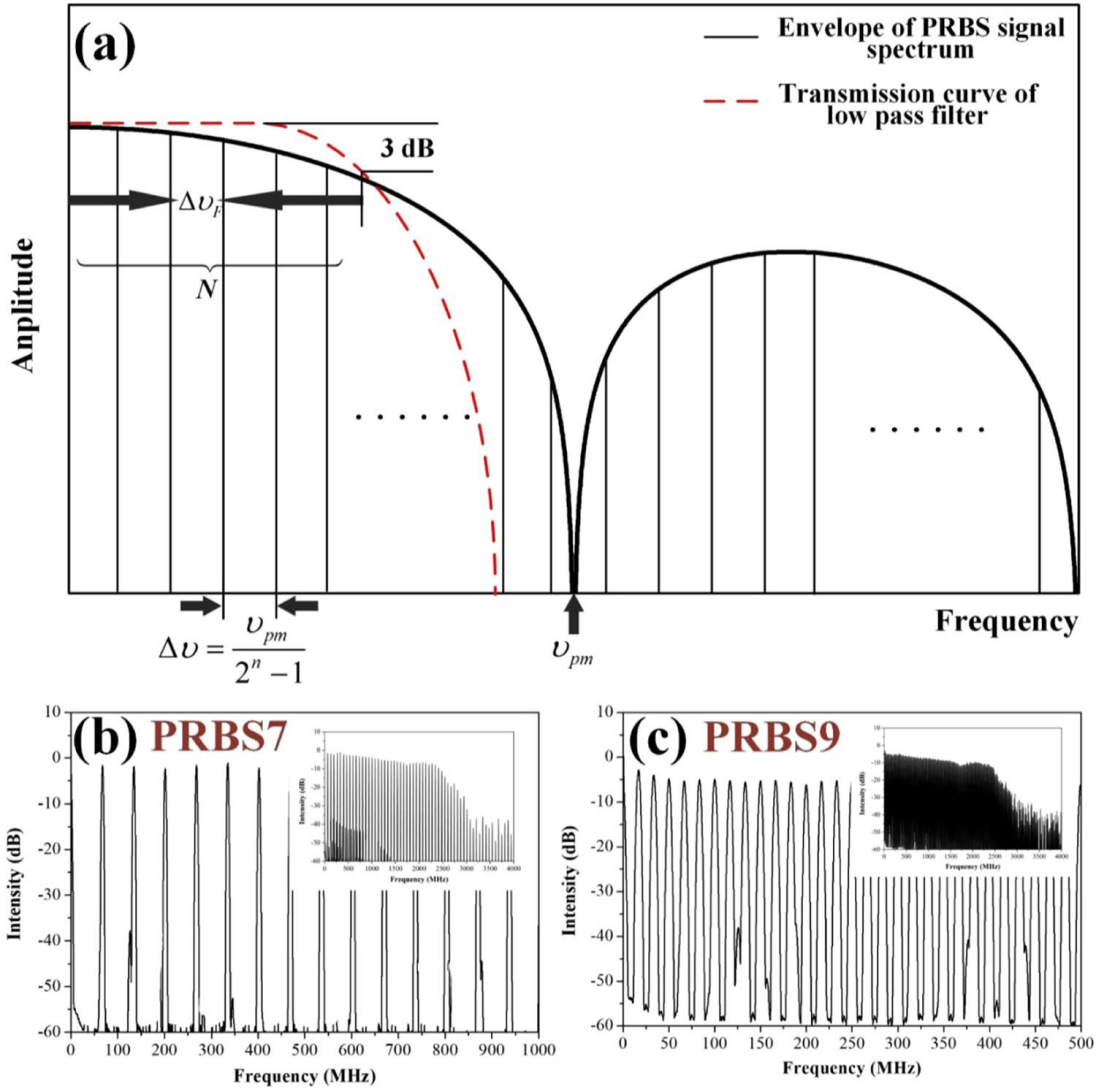
(**a**) Spectrum of the PRBS modulated, low pass filtered phase modulated signal. PRBS spectrum exhibits a periodic and discrete optical frequency comb (black vertical line) within sinc^2^ envelope (black solid curve), transmission curve of low-pass filter exhibits a window with a 3 dB width of Δυ_*F*_ (red dotted curve), the number of spectral lines in the window is *N*; (**b**) the measured spectrum of the PRBS7 modulated, 2.2 GHz low pass filtered RF signal with modulation frequency of 8.5 GHz (insert: wide spectrum of 4 GHz); (**c**) the measured spectrum of the PRBS9 modulated, 2.2 GHz low pass filtered RF signal with modulation frequency of 8.5 GHz (insert: wide spectrum of 4 GHz).

A triply coupled, set of nonlinear partial differential equations can be used to characterize the evolution of laser fields in the SBS suppression system^4,11,16^. In these equations, optical fibers are seeded with phase-modulated light, and the three-wave interaction among the pump (*A*_*L*_), the Stokes (*A*_*S*_), and acoustic (*ρ*) fields is described as following:

1 $$\frac{c}{n}\frac{\partial {A}_{L}}{\partial z}+\frac{\partial {A}_{L}}{\partial t}=\frac{i\omega {\gamma }_{e}}{2{n}^{2}{\rho }_{0}}\rho {A}_{S}$$

2 $$-\frac{c}{n}\frac{\partial {A}_{S}}{\partial z}+\frac{\partial {A}_{S}}{\partial t}=\frac{i\omega {\gamma }_{e}}{2{n}^{2}{\rho }_{0}}{\rho }^{\ast }{A}_{L}$$

3 $$\frac{\partial \rho }{\partial t}+\frac{{\Gamma }_{B}}{2}\rho =\frac{i{\varepsilon }_{0}{\gamma }_{e}{q}^{2}}{2{\Omega }_{B}}{A}_{L}{{A}_{S}}^{\ast }+f$$

here *γ*_*e*_ is the electrostrictive constant, Г_*B*_ is the Brillouin bandwidth, Ω_*B*_ is the resonant acoustic frequency, *c* is the speed of light, *n* is the refractive index of fiber, *ω* is the optical frequency (*ω ≈ ω*_*L*_ *≈ ω*_*S*_), *ω*_*L*_ is the frequency of the pump light, which is the signal light of amplifier, *ω*_*S*_ is the frequency of the Stokes light, *ρ*_0_ is the mean density of the fiber medium, *ε*_0_ is the vacuum permittivity, and *f* is a Gaussian random variable which initiates SBS. These equations can be solved via the initial conditions of the phase modulated pump wave^7,11,12^. When the optical spectrum of the pump laser is modulated to generate a series of equidistant spectral lines, the effective Brillouin gain *G*_*eff*_ is expressed as^17^

4 $${G}_{eff}({\omega }_{S,n})={g}_{0}({\Gamma }_{B}/2){L}_{eff}\mathop{\sum }\limits_{m=-N}^{N}\frac{{I}_{L,m}}{{({\Gamma }_{B}/2)}^{2}+{[{\omega }_{L,m}-{\omega }_{S,n}-{\Omega }_{a}]}^{2}},$$

where *L*_*eff*_ is the effective length of the fiber; *g*_0_ = *γ*_*e*_^2^*ω*^2^/*ρ*_0_*nc*^3^*ν*_*S*_Г_*B*_; *ω*_*L,j*_ = *ω*_*L*,0_ + *j*Δυ, *ω*_*S,j*_ = *ω*_*S*,0_ + *j*Δυ; *ω*_*L*,0_ is the central frequency of the pump light, Ω_a_ is the frequency of the acoustic wave resonant with the laser mode oscillating at *ω*_*L*,0_; *I*_*L,m*_ is the intensity of the pump light. The phase modulation is implemented by using a PRBS signal to drive the electro-optic modulator. The spectral line spacing depends on modulation frequency and pattern length,

5 $$\Delta \upsilon =\frac{{\upsilon }_{pm}}{{2}^{{\rm{n}}}-1},$$

where, *υ*_*pm*_ is the modulation frequency of PRBS, n is the pattern length. The spectrum of PRBS presents sinc^2^-function envelope and the linewidth of the pump laser is determined by the modulation frequency^7^. Based on Eq. (5) continuously adjusting the spectral line spacing can only be achieved by controlling the modulation frequency of PRBS signal generator. Therefore, a special spectral line spacing and a narrow pump linewidth becomes a dilemma due to the correlation of the modulation frequency of the unfiltered PRBS RF signal. One attractive approach to manipulate pump linewidth is to filter the PRBS signal with a low-pass filter. In Fig. 4(a), the modulated pump linewidth is determined by the bandwidth of the low-pass filter (Δυ_*F*_). The spectrum of modulated pump laser presents a frequency comb with the envelope of

6 $${I}_{L,m}=A\,\sin \,{c}^{2}(\frac{m\Delta \upsilon }{{\upsilon }_{pm}}\pi ),$$

where *A* is the modulation coefficient. Substituting Eq. (6) to Eq. (4), the Brillouin gain modulated by PRBS is

7 $${G}_{eff}({\omega }_{S,n})=\frac{{g}_{0}({\Gamma }_{B}/2){L}_{eff}}{\mathop{\sum }\limits_{m=-N}^{N}\sin \,{c}^{2}(\frac{m\Delta \upsilon }{{\upsilon }_{pm}}\pi )}\mathop{\sum }\limits_{m=-N}^{N}\frac{\sin \,{c}^{2}(\frac{m\Delta \upsilon }{{\upsilon }_{pm}}\pi )}{{({\Gamma }_{B}/2)}^{2}+{[2\pi (m-n)\Delta \upsilon ]}^{2}},$$

where *N* is equal to Δυ_*F*_/2Δυ. The intensity distribution of optical modes has been normalized, the *G*_*eff*_ is a convolution of the laser spectrum and the spontaneous Brillouin linewidth^12^. Therefore, a certain value of *n* can be found to derive a maximum Brillouin gain

8 $${G}_{eff,\max }=\frac{{g}_{0}({\Gamma }_{B}/2){L}_{eff}}{\mathop{\sum }\limits_{m=-N}^{N}\sin \,{c}^{2}(\frac{m\Delta \upsilon }{{\upsilon }_{pm}}\pi )}\mathop{\sum }\limits_{m=-N}^{N}\frac{\sin \,{c}^{2}(\frac{m\Delta \upsilon }{{\upsilon }_{pm}}\pi )}{{({\Gamma }_{B}/2)}^{2}+{[2\pi (m-{n}_{\max })\Delta \upsilon ]}^{2}}$$

Generating the strongest Stokes light, the maximum Brillouin gain determines the SBS threshold. Consequently, the SBS threshold enhancement factor can be expressed by

9 $$\frac{{P}_{th}}{{P}_{0}}=\frac{{G}_{eff,\max }}{{G}_{eff,0}}=\frac{\mathop{\sum }\limits_{m=-N}^{N}\sin \,{c}^{2}(\frac{m\Delta \upsilon }{{\upsilon }_{pm}}\pi )}{{({\Gamma }_{B}/2)}^{2}\mathop{\sum }\limits_{m=-N}^{N}\frac{\sin \,{c}^{2}(\frac{m\Delta \upsilon }{{\upsilon }_{pm}}\pi )}{{({\Gamma }_{B}/2)}^{2}+{[2\pi (m-{n}_{\max })\Delta \upsilon ]}^{2}}},$$

where *G*_*eff*, 0_ = 2*g*_*0*_*L*_*eff*_/Г_*B*_ is the Brillouin gain of a single-frequency pump laser^12^.

**Figure 5:**
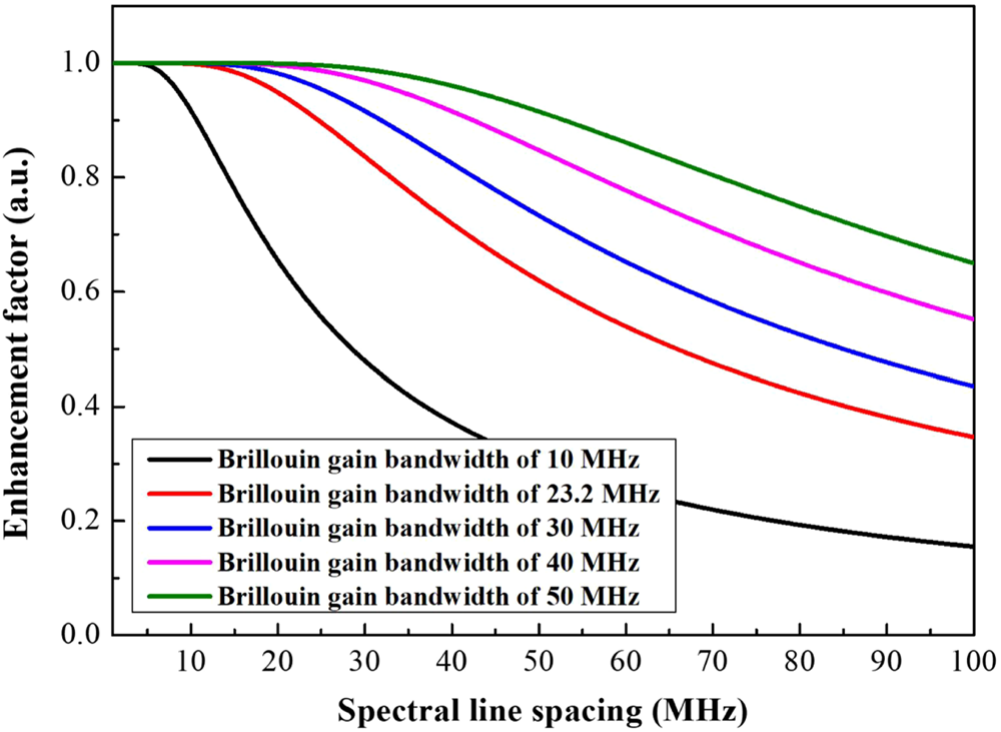
Enhancement factor as functions of spectral line spacing for five Brillouin gain bandwidth with a filter bandwidth of 2.2 GHz and PRBS modulation frequency of 6.5 GHz.

In order to calculate the SBS threshold enhancement factor based on Eq. (9), we measured the Brillouin gain spectrum of the fiber amplifier, as drawn in Fig. 3. The normalized enhancement factors were simulated with Г_*B*_ = 1.46 × 10^8^ rad/s (corresponds to Brillouin gain bandwidth of 23.2 MHz), Δυ_*F*_ = 2.2 GHz, and *υ*_*pm*_ = 6.5 GHz. Subsequently, the Brillouin gain bandwidth was expanded to different bandwidths varying from 10 MHz to 60 MHz is shown in Fig. 5. As is shown, the threshold enhancement factor is in an inverse proportion to the spectral line spacing and appears to a saturation character, and there is a strong dependence on the Brillouin gain bandwidth for the saturation values and the plateaus (maximizing threshold enhancement factor). For a given linewidth, the number of spectral line (*N*) is inversely proportional to the spectral line spacing, as shown in Fig. 4(a). More spectral lines make the optical spectral shape homogeneous as presented in Fig. 7, which will improve the SBS threshold. When the spectral line spacing is further reduced, the convolution of the Brillouin gain spectrum and the spectral line is increased according to the number of the spectral line *N* in the Eq. (9), which ultimately offsets the threshold enhancement caused by spectral homogenization and reaches plateau. Thus, a certain spectral line spacing can be found to maximize threshold enhancement factor. Simultaneously, a large Brillouin gain bandwidth causes the plateau occur at a large spectral line spacing due to the extension in the overlap range.

**Figure 6:**
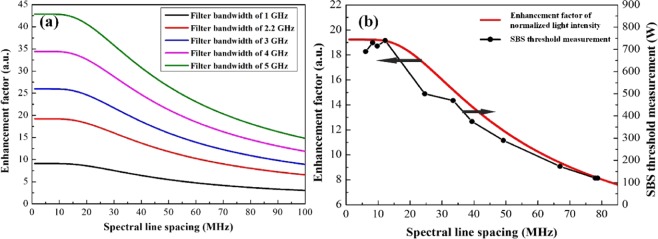
(**a**) Enhancement factor as functions of spectral line spacing for five filter bandwidth with a Brillouin gain bandwidth of 23.2 MHz and PRBS modulation frequency of 6.5 GHz; (**b**) comparison of trend between enhancement factor of normalized light intensity (red curve) and SBS threshold measurements (black circle) in filter bandwidth of 2.2 GHz case.

**Figure 7:**
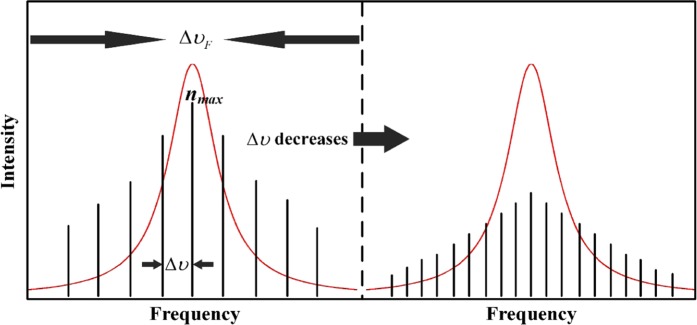
Schematic of the evolution as the spectral line spacing decreases. The Brillouin gain bandwidth (red curve) is constant and the spectrum (black vertical line) is homogenized during this process.

Based on the above analysis, we simulated the normalized enhancement factor for different filter bandwidths varying from 1 GHz to 5 GHz, as plotted in Fig. 6(a). The Brillouin gain bandwidth and PRBS modulation frequency were maintained at 23.2 MHz and 6.5 GHz, respectively. As is shown, the saturation values have a strong dependence on the filter bandwidth, and there is an apparent irrelevance in the filter bandwidth for the plateaus. A wider filter bandwidth transmits more spectral lines with a same spectral line spacing, which more effectively homogenizes the spectrum and enhances the SBS threshold. For different filter bandwidths, the overlap range of the Brillouin gain spectrum and the spectral line almost stays a constant since the Brillouin gain bandwidth is much smaller than the filter bandwidth, as shown in Fig. 7. In this case, the spectral line spacing of the plateaus is also constant. Based on 1.2 kW four-stage monolithic fiber amplifier shown in Fig. 1, we measured the SBS thresholds in the case of 2.2 GHz filter bandwidth, and compared with the simulated data in Fig. 6(b). In our experiment, the intensity of the separated spectral line was controlled to be nearly the same level. The SBS threshold decline trend represent good agreement with the simulation. When the spectral line spacing was reduced to about 10 MHz, the SBS threshold slightly fluctuated with varying the spectral line spacing. This result adjacent to the estimate (9.6 MHz) of the plateau in Fig. 6(a). Similar results had been demonstrated in^4,11^.

We chose a spectral line spacing (12.7 MHz) close to the plateau to modulate DFB diode laser and DBR laser seeds, respectively. Due to the inherent current noise, the linewidth of DFB laser is generally wider than that of DBR laser^18,19^. The spectrum of DFB laser is broadened relative to DBR laser. For current noise broadened DFB laser with a linewidth less than the spectral line spacing, the spectral broadening resulting from PRBS phase modulation and current noise is independent of each other. This is expected, a more homogenous spectrum will be obtained by driving two spectral broadening mechanisms, while the SBS threshold is also enhanced. As is shown in Fig. 2, the SBS threshold was further enhanced when the amplifier was seeded with a DFB laser. However, significant ASE was observed with 1082 nm DFB diode laser wavelength due to the strong emission cross section of the Yb-doped fiber around 1070 nm.

## Methods

The schematic diagram of PRBS modulated, low pass filtered, Yb-doped monolithic fiber amplifier is shown in Fig. 1. DFB diode laser (1075 nm) and DBR laser (1082 nm) were used to seed the amplifier, respectively. A PRBS generator and a low-pass RF filter served to generate pseudo-random binary sequence signal of a specific bandwidth and arbitrary spectral line spacing. Subsequently, RF signals were magnified by a ~25 dB RF amplifier. The effective linewidth of the seed was broadened via a fiber-coupled LiNbO_3_ electro-optic modulator. Thereafter, a third-stages amplifier was implemented to amplify the broadened seed to ~12 W. In the main amplifier stage, six 300 W (976 nm) fiber-coupled laser diodes were combined by a (6 + 1) × 1 pump/signal combiner, a 7 m-long non-polarization maintaining 20/400 μm Yb-doped fiber (manufactured by nLight) was utilized to be the gain medium. A high power cladding stripper (CPS) was installed to eliminate the redundant light in the cladding. At the amplifier output, the pigtail fiber was cleared to 8° to avoid unexpected end reflection. The circulator was applied between pre amplifier stage and main amplifier stage to protect the preceding stage and monitor backward scatting light. Different spectral line spacings were implemented while maintaining the filter bandwidth of 2.2 GHz. For each spectral line spacings, the intensity of each phase modulated signal was maintained to be the same level as drawn in Fig. 4(b,c). The seed was amplified to 1.2 kW by driving a phase modulated signal with modulation frequency of 6.5 GHz and pattern length of 9, the modulation linewidth of the modulated signal was limited by a low-pass RF filter with 3 dB bandwidth of 2.2 GHz.

The single-frequency laser (1075 nm) operated in the amplifier without phase modulation in order to measure single-frequency SBS threshold. The output power of amplifier was measured through 1.5 kW range thermal power meter. A 5 W range integrating sphere power meter was used to measure the power of backward scatting light. The spectral content of forward light and backward scatting light was sampled utilizing an optical spectrum analyzer (OSA) of 0.02 nm resolution bandwidth. The spectrum of the phase modulated signal was measured through a wideband (10 Hz to 26.5 GHz) spectrum analyzer (KEYSIGHT N9020A), a 5 dB coaxial attenuator was placed at the input of the spectrum analyzer for overpower protection. The beam quality of the output power at 1 kW was measured by the LQM-20 (@PRIMES Corporation) using four-sigma method. The temperature of the fiber amplifier system was maintained at 20 °C by water cooler, at the same time, the temperature of the high power CPS was kept within 60 °C by a dedicated water cooler.
